# Hypobaric Hypoxia Ameliorates Impaired Regeneration After Diabetic Skeletal Muscle Injury by Promoting HIF-1α Signaling

**DOI:** 10.3390/ijms27020648

**Published:** 2026-01-08

**Authors:** Jinrun Lin, Minghao Geng, Li Zhou, Danni Qu, Hao Lin, Jihao Xing, Ryosuke Nakanishi, Hiroyo Kondo, Noriaki Maeshige, Hidemi Fujino

**Affiliations:** Department of Rehabilitation Science, Graduate School of Health Sciences, Kobe University, 7-10-2, Tomogaoka, Suma-ku, Kobe 654-0142, Japan

**Keywords:** hypobaric hypoxia, diabetes-induced muscle injury, HIF-1α, angiogenesis, vascular remodeling

## Abstract

Diabetes mellitus severely impairs skeletal muscle regeneration after injury, limiting satellite cell activation and angiogenesis and disrupting barrier integrity while increasing fibrosis. Hypobaric hypoxia has been proposed to improve the regenerative microenvironment through hypoxia-responsive signaling, but its temporal effects and the coordination between vascular and myogenic programs in diabetic muscle remain unclear. To clarify these processes, adult male mice were divided into five groups: diabetes mellitus control (DM), cardiotoxin-injured (CTX) diabetes assessed on days 7 and 14 (CTX7, CTX14), and hypobaric-hypoxia-treated diabetic injury assessed on days 7 and 14 (H+CTX7, H+CTX14). Animals in the hypoxia groups were exposed to a hypobaric hypoxia chamber for 8 h per day for 14 days. Fibrosis, angiogenic and myogenic markers, and endothelial junctional genes were examined using histology, immunofluorescence, immunoblotting, and qRT-PCR (Quantitative Real-Time PCR). Hypobaric hypoxia on day 7 enhanced HIF-1α (hypoxia-inducible factor 1 alpha), VEGF (vascular endothelial growth factor), eNOS (endothelial nitric oxide synthas), Kdr (kinase insert domain receptor, VEGFR-2), and Angpt2 (angiopoietin-2) expression, accompanied by simultaneous endothelial sprouting and early myogenic stimulation compared to CTX7. Improvements were observed in Angpt1 (angiopoietin-1), Cdh5 (cadherin-5, VE-cadherin), Emcn (endomucin), the Angpt1/Angpt2 ratio, and CD31 density. Myogenin and MyHC (myosin heavy chain) were induced with a reduction in eMyHC (embryonic myosin heavy chain) in accordance with stabilization of endothelium and maturation of fibers, which occurred by day 14. A decrease in fibrosis and an increase in the myofiber cross-sectional area occurred. These findings suggest that hypobaric hypoxia modulates HIF-1α signaling, which in turn induces the VEGF-Kdr-eNOS pathway and the angiopoietin–Tie2–VE-cadherin pathway. Together, these pathways coordinate vascular remodeling and myogenic regeneration, ultimately improving the structural and functional recovery of diabetic muscle.

## 1. Introduction

Along with its established cardiac, renal, and neurological morbidity, diabetes significantly impairs skeletal muscle healing following injury. Reduced capillary density, inhibited hyperemic responsiveness, and hyporesponsive pro-angiogenic signaling comprise the key pathology of microcirculatory damage and defective angiogenesis in patients [1]. Diabetic subjects and animal models typically exhibit an impaired progression of inflammatory clearance to the myogenic program, ultimately preventing maturation and remodeling [1,2]. Chronic hyperglycemia, insulin resistance, and systemic inflammation continue to inhibit satellite cell activation and differentiation, misdirect the angiogenic response, and facilitate tissue rebuilding [1,3]. These cascading malfunctions appear in the form of slow structural and functional recovery, reduced mobility, and quality of life [1].

Angio-myogenesis is an intrinsic process closely associated with skeletal muscle regeneration, characterized by the coordinated formation of new blood vessels and muscle fibers. Genetic or pharmacological inhibition of VEGF reduces baseline capillarization and impairs angiogenic responses during exercise or tissue repair [4]. Secondly, local perfusion can be improved by restoring the VEGF axis. This is achieved by employing gene transfer or protein delivery to stimulate regeneration [5]. These results, when combined, provide a paradigm whereby VEGF-controlled capillary support is essential to achieve the best myogenic results [4,5]. Simultaneously, endothelium-generated signals also act on muscle progenitors; several studies report that endothelial paracrine ligands such as *VEGF* serve in a feedback manner to control satellite cell behavior and myogenesis development [2,6]. These findings indicate that the remodeling processes of capillaries and myogenesis occur together rather than as part of a sequence. Following muscle damage, eMyHC-positive nascent myofibers accumulate around day 7, which is the point of activation, differentiation, and primary fusion. Fibers transition to mature MyHC expression and begin structural and functional remodeling by day 14 [7]. Thus, the choice of days 7 and 14 as the points of observation reflects early vascular support to myogenesis as well as the subsequent role MyHC plays in maturation in a common time framework [8].

Hypobaric hypoxia can enhance HIF-1α and VEGF signaling; promote capillary remodeling; and synergize with muscle contraction and exercise [9,10,11,12]. In diabetes, excessive oxidative stress and metabolic toxicity destabilize HIF-1α and decrease its transcriptional activity. This leads to the downregulation of VEGF and related targets and capillary rarefaction, which further decouples angio-myogenesis [13,14,15]. Metabolic repurposing of HIF-1α, a protein stable in low oxygen, is an effective method for hypoxia-induced angiogenesis, restructuring metabolism, and repairing tissue by directly activating VEGF and Angpt2 in endothelium. The hypoxic sublamar niche of satellite cells suggests that genetic data from both HIF-1α and HIF-2α are needed to regulate self-renewal and regenerative activities under hypoxia or injury stress, as well as their modest roles during normoxia [16,17,18,19,20].

Based on this, it is anticipated that restoration and rhythmic activation of the HIF-1α axis by intermittent or chronic hypobaric hypoxia will re-coordinate hypoxic signaling with VEGF-dependent angiogenesis and metabolic support, thereby enhancing inflammatory resolution, satellite cell differentiation, tissue remodeling, and ultimately, structural and functional recovery [16,17,18]. Compared to growing evidence that intermittent hypobaric hypoxia improves vascular remodeling and exercise adaptations, direct evidence of its effects on diabetic muscle post-injury angiogenesis and myogenesis is scarce [1,9].

We, therefore, hypothesize that exposing diabetic mice with skeletal muscle injury to a hypobaric hypoxia chamber will promote post-injury angiogenesis and myogenesis.

## 2. Results

### 2.1. Establishment of the Type 2 Diabetes Mouse Model

**Figure 1:**
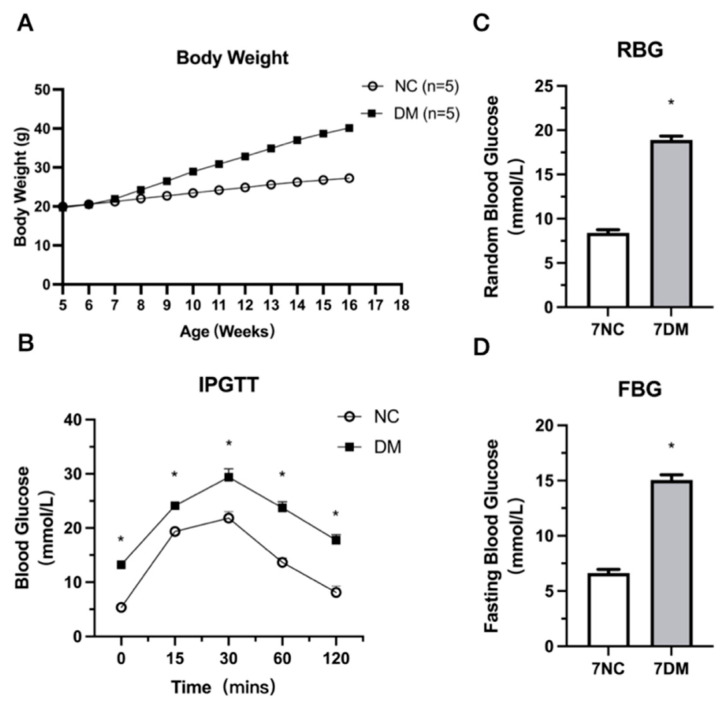
Weight trajectories for mice with normal conrtol (NC) and high-fat diets (HFDs) (**A**). Intraperitoneal glucose tolerance test for blood glucose (**B**). Non-fasting glucose (**C**). Fasting glucose (**D**). Data are shown as mean ± SEM (*n* = 5). NC: normal mice; DM: diabetic mice. * Significant differences exist in the groups of 7NC and 7DM (*p* < 0.05).

Figure 1A shows that, after 12 weeks of a high-fat diet supplemented with streptozotocin (HFD + STZ), DM mice gained more body mass than NC (Normal Control) mice. Non-fasting blood glucose was greater than in NC (Figure 1B). Figure 1C,D show that the DM group had considerably higher glucose tolerance and fasting blood glucose levels than the NC group.

### 2.2. Hypobaric Hypoxia Enlarges the Regenerated Area and Attenuates Fibrosis After Skeletal Muscle Injury

**Figure 2:**
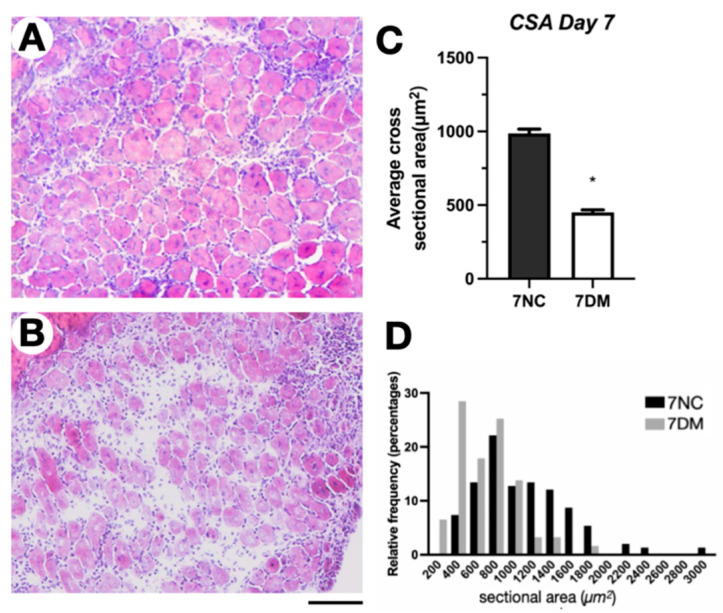
Skeletal muscle H&E staining normal mice at 7 days post-injury (7 dpi) (**A**). 7DM diabetic mice H&E staining was 7 days post-injury (7 dpi) (**B**). Mean myofiber cross-sectional area (CSA) (**C**). Frequency distribution of myofiber CSA (**D**). Bar = 100 µm. Statistics are mean ± SEM (*n* = 5). 7NC: mice that were 7 days old and normal after injury; 7DM: diabetic mice that were 7 days old after injury. * Indicates a significant difference between 7NC and 7DM (*p* < 0.05).

To define the differences in post-injury repair between diabetic and normoglycemic mice, H&E (hematoxylin and eosin) staining was performed on day 7 after injury. Figure 2A,B shows a clear inhibition of regeneration in diabetes patients. Diabetes significantly altered the frequency of distribution for myofiber cross-sectional area (Figure 2C), and quantitative measurement showed worse regeneration in diabetic mice compared to normoglycemic controls (Figure 2D). Furthermore, observations of H&E staining sections revealed that compared with the CTX group, the H+CTX group had a greater number of regenerated muscle fibers containing central nuclei at 7 and 14 days post-injury. This was consistent with the observed increase in muscle fiber cross-sectional area (CSA) and reduction in fibrosis, which together indicate that hypoxia treatment promoted the effective regeneration and mature structural integration of muscle fibers.

**Figure 3:**
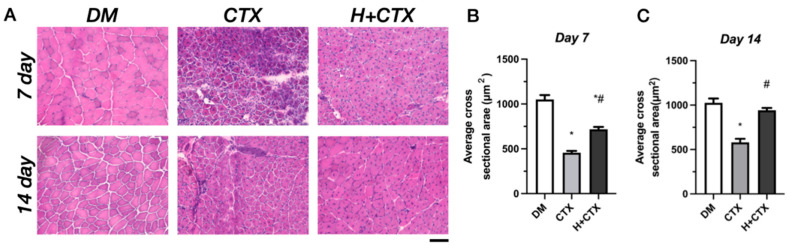
Hematoxylin and eosin (H&E) staining of skeletal muscle in diabetic mice on days 7 and 14 post-injury (**A**). Mean myofiber cross-sectional area (CSA) 7 days post-injury (**B**). Mean myofiber CSA 14 days post-injury. Data are mean ± SEM (*n* = 5) (**C**). Bar = 100 µm. DM: diabetic mice without injury; CTX: diabetic muscle injury; H+CTX: hypobaric-hypoxia-treated diabetic muscle injury. * Indicates the difference from DM; ^#^ indicates the difference between the CTX and H+CTX groups (*p* < 0.05).

We then applied hypobaric hypoxia and collected tissues on days 7 and 14 after injury. On H&E staining, the CTX group displayed extensive necrotic fibers and inflammatory infiltration on day 7 (Figure 3A). Both CTX- and hypobaric-hypoxia-treated injured muscle differed significantly from the diabetic baseline (Figure 3B). In comparison to the CTX-treated group, the hypobaric-hypoxia group showed a pronounced reduction in necrosis and infiltration, indicating partial restoration of regeneration. On day 14, regeneration in CTX was impaired compared to the diabetic baseline (Figure 3C). The hypobaric-hypoxia group approximated normal levels (*p* = 0.2691 versus diabetic baseline) and underwent significant improvements compared to CTX at the same time point, supporting a pro-regenerative effect of hypobaric hypoxia in diabetes.

**Figure 4:**
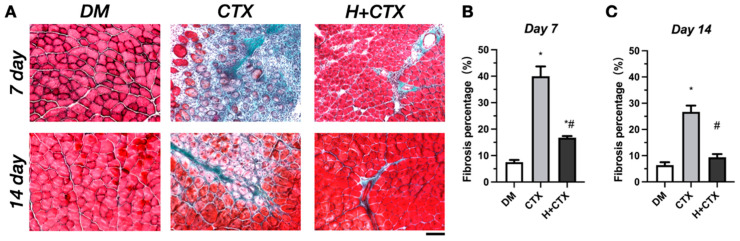
Masson’s trichrome staining of skeletal muscle in diabetic mice on days 7 and 14 post-injury (**A**). Average fibrotic area 7 days after damage (**B**). The average fibrotic area 14 days post-injury (**C**). Bar = 100 µm. Data are shown as mean ± SEM (*n* = 5). DM: diabetic mice without injury; CTX: diabetic muscle injury; H+CTX: hypobaric-hypoxia-treated diabetic muscle injury. * Indicates a significant difference relative to DM; ^#^ indicates a significant difference between CTX and H+CTX (*p* < 0.05).

The trichrome staining used by Masson produces concordant structural readouts (Figure 4A). On day 7, CTX was characterized by a high level of interstitial fibrosis due to a higher amount of endomysial and peri-muscular collagen and expanded septa; the fibrosis was significantly different compared to the diabetic baseline (Figure 4B). The hypobaric-hypoxia group had less interstitial fibrosis compared to CTX. Figure 4C demonstrates that CTX had more fibrosis at day 14 than the control with diabetes, whereas the hypobaric hypoxia group did not (*p* = 0.4444).

A comparison revealed that there was little collagen deposition in the hypobaric-hypoxia group. This level returned to near-control range, with significantly less fibrosis compared to CTX. These results suggest that hypobaric hypoxia counteracts the excessive fibrosis in the diabetic injured muscle and enhances tissue architecture.

### 2.3. Hypobaric Hypoxia Promotes the Expression of Angiogenic Factors Through Stabilization of HIF-1α

**Figure 5:**
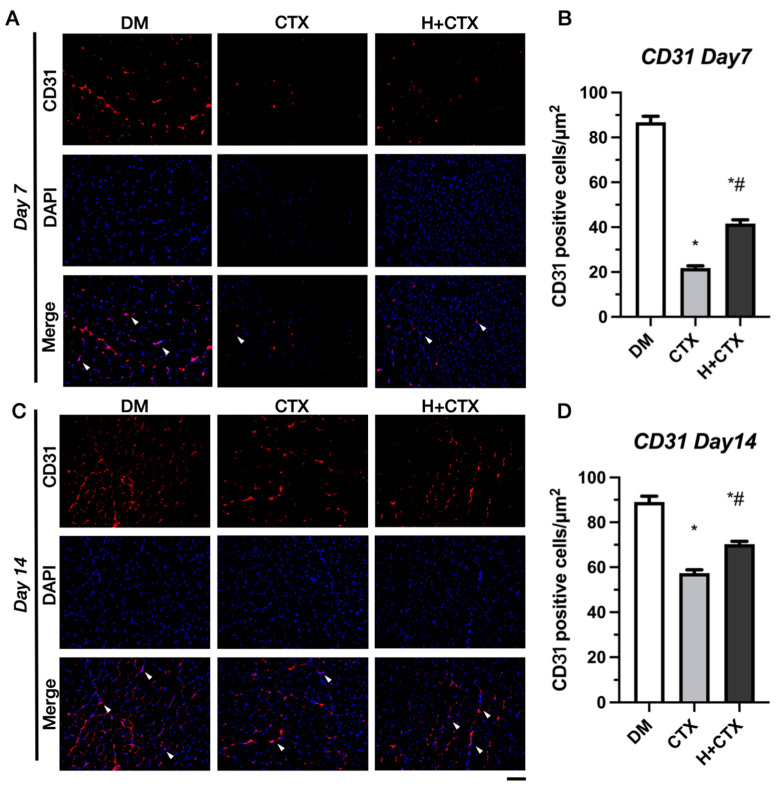
Post-injury CD31 immunofluorescence on day 7 (**A**). Post-injury CD31-positive cell numbers on day 7 (**B**). CD31 immunofluorescence of injury on day 14 (**C**). CD31-positive cell numbers on day 14 post-injury. Data are mean ± SEM (5 per group) (**D**). The white arrowheads indicate CD31 positive cells. Bar =100 µm. DM: diabetic mice without injury; CTX: diabetic muscle injury; H+CTX: hypobaric hypoxia-treated diabetic muscle injury. * Indicates the difference from DM; ^#^ indicates a significant difference between CTX and H+CTX (*p* < 0.05).

On day 7 (Figure 5A), the DM group exhibited the highest density of CD31-positive cells. The CTX group was much smaller than DM (Figure 5B), which means that microvascular damage was prominent immediately after injury. H+CTX was greater than CTX, resulting in an early injury response; however, these levels were lower in DM. CTX was partially recovered on day 14 (Figure 5C), but remained lower than DM (Figure 5D). H+CTX was also higher than CTX but did not reach the DM level. H+CTX outperformed the time-matched CTX groups at both time points, which is in line with a cumulative pro-angiogenic effect.

**Figure 6:**
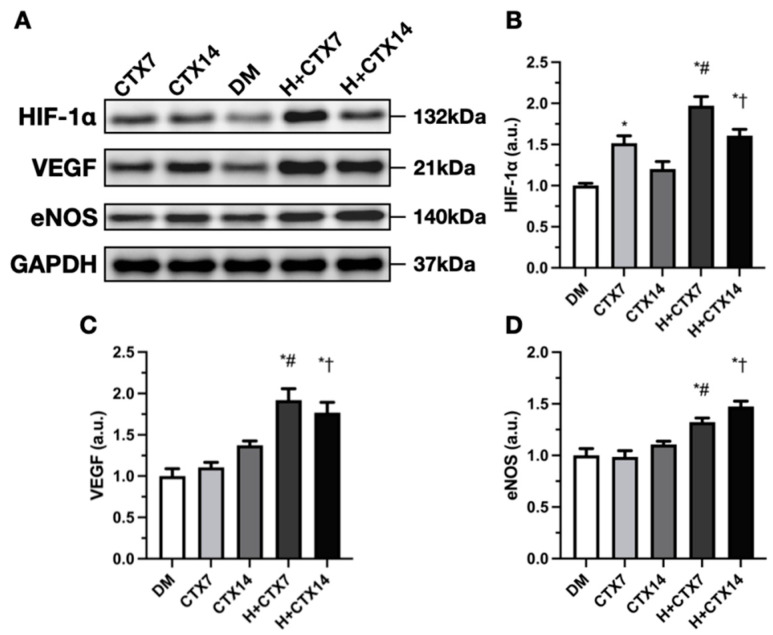
HIF-1a, VEGF, and eNOS Western blots of tibialis anterior muscle (**A**). Protein levels of average groups HIF-1a (**B**), VEGF (**C**), and eNOS (**D**). Data are mean ± SEM (*n* = 5). DM: diabetic mice with no injury; CTX7: diabetic muscle injury on day 7; CTX14: diabetic muscle injury on day 14; H+CTX7: hypobaric-hypoxia-treated diabetic muscle injury on day 7; H+CTX14: hypobaric-hypoxia-treated diabetic muscle injury on day 14. * Indicates the difference from DM; ^#^, ^†^ indicates a significant difference between CTX7 and H+CTX7 and CTX14 and H+CTX14 respectively (*p* < 0.05).

Western blotting bands of protein HIF-1a are shown in the figure below (Figure 6A). Compared to the non-intervention groups, HIF-1a levels in the hypobaric hypoxia groups were significantly elevated (Figure 6B; CTX7 vs. H+CTX7 and CTX14 vs. H+CTX14, respectively). Compared to the DM group, CTX7, H+CTX7, and H+CTX14 showed significant differences, whereas CTX14 demonstrated no significant difference (*p* = 0.4531).

The HIF-1a level in H+CTX was highest on day 7, but decreased on day 14, although it remained higher than that of the same non-intervention group. HIF-1a protein expression was stabilized in the non-intervention group through treatment with hypobaric hypoxia. VEGF proteins are represented below (Figure 6A). Compared to DM, H+CTX7 and H+CTX14 showed substantial increases (Figure 6C), whereas CTX7 and CTX14 did not (*p* = 0.9347 and *p* = 0.0933, respectively).

The hypobaric hypoxia groups had higher values than the time-matched non-intervention groups (*p <* 0.05) at both time points, indicating that VEGF was highest on day 7 following intervention. It then decreased slightly on day 14 despite maintaining high overall elevation. Figure 6A shows the complex processes of eNOS proteins. CTX7 and CTX14 did not vary compared to DM (Figure 6D, *p* = 0.9996 and *p* = 0.5898). Hypobaric hypoxia significantly boosted eNOS protein expression. The H+CTX7 group had higher expression levels than the DM group and matched CTX’s timing; H+CTX14 increased further, exceeding the DM and the time-matched CTX. Overall, the results of eNOS showed an immediate rise after intervention, with the highest level found on day 14.

**Figure 7:**
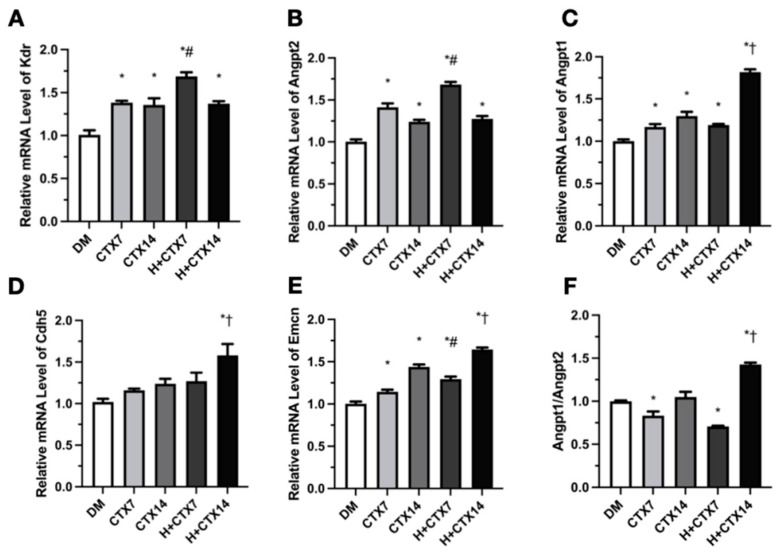
Quantitative analysis of angiogenesis-related mRNA expression. Kdr (**A**). Angpt2 (**B**). Angpt1 (**C**). Cdh5 (**D**). Emcn (**E**). Angpt1/Angpt2 ratio (**F**). Data represent mean ± SEM (*n* = 5 per group). DM: diabetic mice without injury; CTX7: diabetic muscle injury on day 7; CTX14: diabetic muscle injury on day 14; H+CTX7: hypobaric-hypoxia-treated diabetic muscle injury on day 7; H+CTX14: hypobaric-hypoxia-treated diabetic muscle injury on day 14. * Indicates the difference from DM; ^#^, ^†^ indicates a significant difference between CTX7 and H+CTX7 and CTX14 and H+CTX14 respectively (*p* < 0.05).

qRT-PCR was used to assess skeletal muscle angiogenesis and endothelial homeostasis transcripts on days 7 and 14 post-injury. The CTX7 and CTX14 groups had moderately but substantially increased Kdr (VEGFR2) expression compared to the DM group (Figure 7A). Kdr expression levels were greater in H+CTX7 and H+CTX14 compared to DM, with H+CTX7 showing the greatest expression. Within time points, Kdr expression was greater in the H+CTX7 and H+CTX14 groups but did not differ substantially (*p* = 0.9996).

Enhancement was prominent on day 7, with a slight decline observed on day 14. Angpt2 expression was higher in the CTX7, CTX14, H+CTX7, and H+CTX14 groups compared to the DM group (Figure 7B). The expression level of H+CTX7 surpassed CTX7 and peaked, but H+CTX14 and CTX14 did not vary (*p* = 0.9485). Angpt1 expression levels in the CTX7, CTX14, H+CTX7, and H+CTX14 groups were greater than in the DM group (Figure 7C). Expression on day 7 was comparable for H+CTX7 and CTX7 (*p* = 0.9864). On day 14, H+CTX14 outperformed CTX14. The DM group had similar Cdh5 (cadherin-5, VE-cadherin) expression to CTX7, CTX14, and H+CTX7 (*p* = 0.7119, *p* =0.2621, *p* = 0.2009, respectively, Figure 7D). However, expression of H+CTX14 was higher than DM. Within time, the H+CTX7 and CTX7 groups attained equal levels (*p* = 0.8721), but the H+CTX14 group showed higher levels. The rise on day 14 indicates stronger junctional/barrier transcription. Emcn (endomucin) expression levels were greater in the CTX7, CTX14, H+CTX7, and H+CTX14 groups compared to the DM group (Figure 7E). H+CTX levels were higher than those of the CTX group at both time periods.

The increase was most evident on day 14, suggesting enhanced endothelial surface-associated transcription. The Angpt1/Angpt2 ratio relative to DM in the H+CTX7 group was lower, and H+CTX14 was highest in each group (Figure 7F). Over time, CTX7 exceeded H+CTX7 without significance (*p* = 0.1414), and H+CTX14 exceeded CTX14. The ratio decreased on day 7 and increased on day 14, which is consistent with an early Angpt2-dominant phase followed by later Angpt1-dominant stabilization.

### 2.4. Hypobaric Hypoxia Promotes the Expression of Muscle-Related Factors

**Figure 8:**
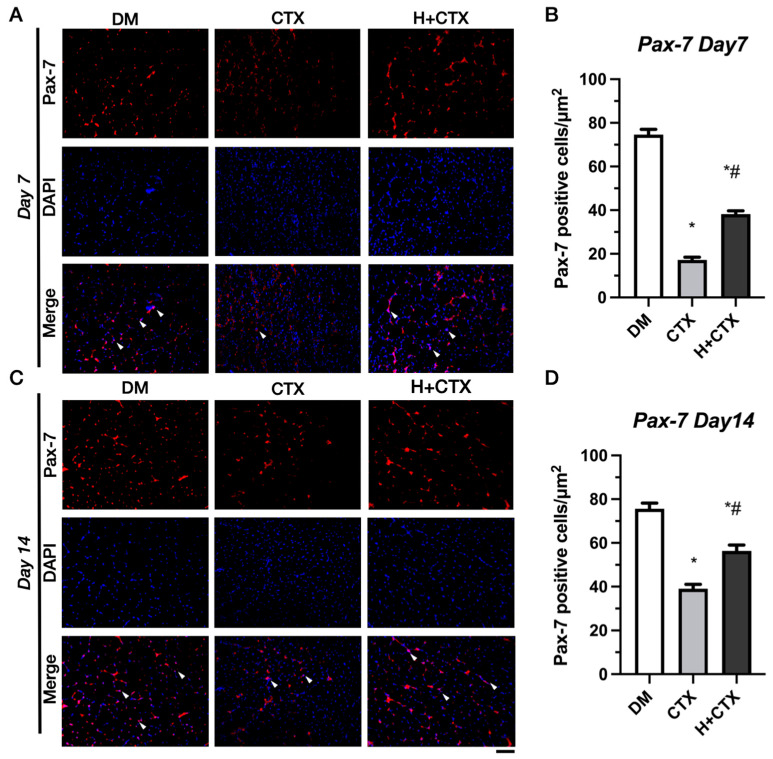
Pax-7 immunofluorescence 7 days post-injury (**A**). Pax-7-positive cell counts 7 days post-injury (**B**). Pax-7 immunofluorescence 14 days post-injury (**C**). Pax-7-positive cell numbers 14 days post-injury (**D**). The white arrowheads indicate Pax-7 positive cells. Bar = 100 µm. Data are mean ± SEM (*n* = 5). DM: diabetic mice without injury; CTX: diabetic muscle injury; H+CTX: hypobaric-hypoxia-treated diabetic muscle injury. * Indicates the difference from DM; ^#^ indicates a significant difference between CTX and H+CTX (*p* < 0.05).

On day 7, the DM group exhibited the highest density of Pax-7-positive cells (Figure 8A). Compared to DM, CTX demonstrated a considerable decrease (Figure 8B). This implies a considerable early decline in the satellite cell pool after damage. On day 7, Pax-7-positive cells in the H+CTX group were considerably greater than in the CTX group but remained lower than in the DM group. This demonstrates an improvement from early satellite cell loss, though only partial recovery was achieved. The CTX group had reduced levels of Pax-7-positive cells on day 14 compared to the DM group (Figure 8C,D). The H+CTX group showed a considerable increase in Pax7-positive cells by day 14, surpassing the CTX group but not reaching levels of the DM group. This indicates that hypobaric hypoxia promotes the replenishment and maintenance of Pax-7-positive satellite cells over time.

**Figure 9:**
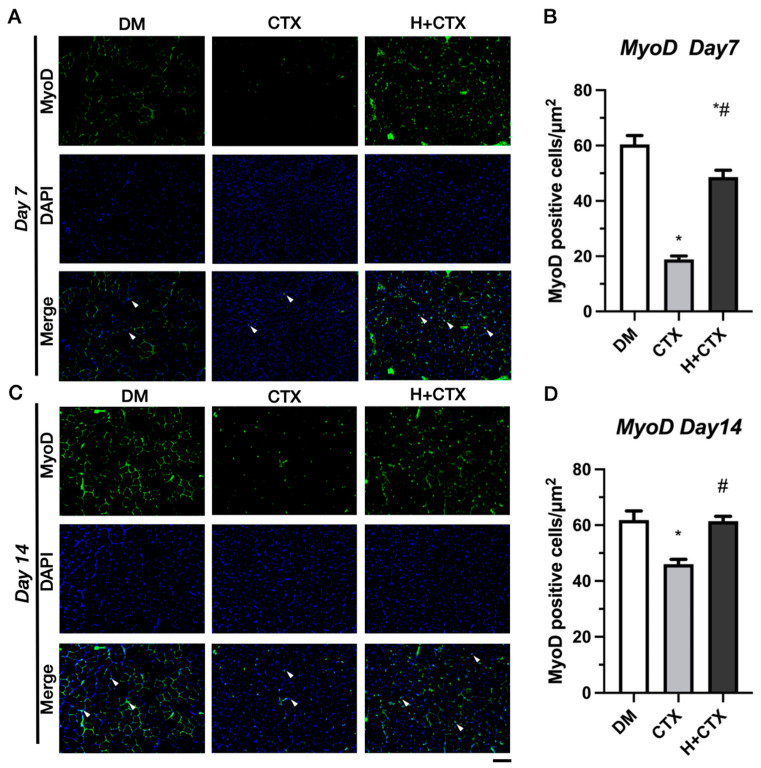
MyoD immunofluorescence 7 days post-injury (**A**). MyoD-positive cell numbers 7 days after damage (**B**). MyoD immunofluorescence 14 days post-injury (**C**). MyoD-positive cell counts 14 days after damage (**D**). The white arrowheads indicate MyoD positive cells. Bar =100 µm. Data are mean ± SEM (*n* = 5). DM: diabetic mice without injury; CTX: diabetic muscle injury; H+CTX: hypobaric-hypoxia-treated diabetic muscle injury. * Indicates the difference from DM; ^#^ a significant difference exists between CTX and H+CTX (*p* < 0.05).

On day 7, the DM group exhibited the highest density of MyoD-positive cells compared to the group with induced muscle injury (Figure 9A). CTX had considerably fewer MyoD-positive cells than DM (Figure 9B).

On day 7, the H+CTX group had significantly more MyoD-positive cells than the CTX group but fewer than the DM group (*p <* 0.05). This indicates an improvement in early satellite cell activation but not DM recovery. On day 14 (Figure 9C), the CTX group had considerably smaller MyoD-positive cells than the DM group (Figure 9D). MyoD-positive cells in the H+CTX group were substantially larger than in the CTX group and reached DM levels (*p* = 0.9923).

**Figure 10:**
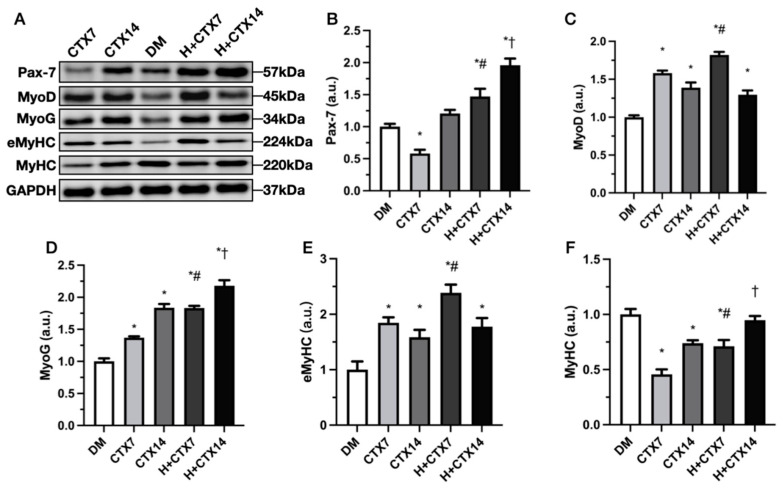
Representative Western blots of Pax-7, MyoD, myogenin (MyoG), eMyHC (embryonic myosin heavy chain), and MyHC (myosin heavy chain) protein levels in the tibialis anterior muscle are presented (**A**). Mean protein expressions of Pax-7 (**B**), MyoD (**C**), MyoG (**D**), eMyHC (**E**), and MyHC (**F**) in each group. Data are mean ± SEM (*n* = 5). DM: diabetic mice without injury; CTX7: diabetic mice with muscle injury on day 7; CTX14: diabetic muscle injury on day 14; H+CTX7: hypobaric-hypoxia-treated diabetic muscle injury on day 7; H+CTX14: hypobaric-hypoxia-treated diabetic muscle injury on day 14. * Indicates *p* < 0.05 and DM; ^#^ shows the difference between CTX7 and H+CTX7; ^†^ indicates a significant difference between CTX14 and H+CTX14 (*p* < 0.05).

The CTX group demonstrated substantially lower Pax-7 protein expression than the DM group on day 7 (Figure 10B), but there was no change by day 14 (*p* = 0.4167). Hypobaric hypoxia had considerably greater levels than DM at both time periods, with the H+CTX group exhibiting the highest value on day 14. At the same time point, the H+CTX group exceeded the CTX group on both days 7 and 14. These findings indicate that hypobaric hypoxia on days 7 and 14 increases or maintains the satellite cell pool, reversing the initial tendency where the CTX7 group was lower than the DM group.

The CTX group had increased MyoD expression on day 7 compared to the DM group, and the H+CTX group exhibited a substantial increase (Figure 10C). On day 14, MyoD expression was greater in the CTX and H+CTX groups compared to DM. On day 7, the H+CTX group exhibited considerably higher MyoD expression compared to the CTX group. Day 14 showed no change between H+CTX and CTX (*p* = 0.6525).

The CTX and H+CTX groups had higher levels of MyoG expression than the DM group on days 7 and 14, with the H+CTX group peaking on day 14 (Figure 10D). On days 7 and 14, the H+CTX group had substantially greater MyoG expression than the CTX group. These results indicate that hypobaric hypoxia enhances differentiation markers by day 7 and further amplifies them by day 14.

On days 7 and 14, the CTX and H+CTX groups showed substantially increased eMyHC expression than the DM group (Figure 10E). The H+CTX category rose to its highest value on day 7 and had significantly higher eMyHC expression than the CTX group. H+CTX showed greater eMyHC expression than CTX on day 14; however, the difference was not significant (*p* = 0.8397). The magnitude of increase had decreased compared to day 7. These findings indicate that hypobaric hypoxia significantly promotes the early formation of regenerative fibers (which is most pronounced at the peak on day 7) and maintains levels exceeding those of the DM group even on day 14.

Compared to DM, both CTX and H+CTX showed significantly lower values on day 7 (Figure 10F). On day 14, CTX maintained a substantially lower value than DM, while H+CTX approached the DM value (*p* = 0.9187). At the same time point, H+CTX and CTX showed significant differences on both days 7 and 14. These results indicate that mature myosin heavy chain is reduced early after injury and gradually recovers to DM levels by day 14. The hypobaric hypoxic environment promotes a more stable recovery of this maturation marker.

## 3. Discussion

This research is the first to show that hypobaric hypoxia accelerates satellite cell proliferation and differentiation in diabetes-related healing from muscle damage.

In a diabetic skeletal muscle injury model, hypobaric hypoxia stabilized and upregulated HIF-1α despite adverse metabolic and microcirculatory conditions. This activation enhanced VEGF and eNOS, promoting mature neovascularization, restoring barrier function through increased Angpt1 and Angpt2 transcripts, and recovering VE-cadherin encoded by Cdh5. Furthermore, markers indicating a continuum from the satellite cell to myocyte—Pax-7, MyoD, and myogenin—and the transition from embryonic to mature myosin heavy chain showed an orderly increase. Histology confirmed clearance of necrosis, regeneration of myofibers, and reduced fibrosis. Together, these findings support the conclusion that hypobaric hypoxia improves angiogenesis and myogenesis post-injury in diabetes by enhancing HIF-1α signaling.

Regeneration in diabetic muscle is limited by two interlinked barriers. These are insufficient activation and expansion of satellite cells, and a deteriorating inflammatory and perfusion environment. Together, these factors delay the window of opportunity for regeneration, limit myotube maturation, and heighten susceptibility to fibrosis [21]. Animal and human studies demonstrate an overall decline in regenerative capacity in diabetes, with depletion and impaired differentiation of the satellite cell pool compounded by angiogenic and endothelial dysfunction [1]. Within a single model, our data showed enhanced vascular and myogenic readouts with phase-specific emphasis, providing empirical support for a coupled-intervention paradigm. On the vascular side, maintaining Tie2 activity, increasing Angpt1, and stabilizing VE-cadherin junctions improve stability and reduce leakage without suppressing angiogenesis [22]. On the myogenic side, Pax-7 is essential for adult regeneration, and the sequential rise in MyoD and myogenin, together with the decline in eMyHC, indicates progression toward mature fibers [23]. In this study, the presence of regenerative muscle fibers containing a central nucleus is a key morphological marker for evaluating the regenerative process. We observed that the hypoxia treatment group (H+CTX) exhibited larger muscle fiber cross-sectional area (CSA) and less fibrosis after injury, which is accompanied by molecular conversion from embryonic myosin heavy chain isoforms (eMyHC) to mature MyHC. These results together form a complete chain of evidence. Hypoxia, by stabilizing the HIF-1α signal, not only promotes the activation of satellite cells indicated by markers Pax-7 and MyoD and the differentiation indicated by MyoG, but also ultimately guides the effective formation, expansion, and structural maturation of new muscle fibers. The latter is directly manifested histologically as the increase in fibers containing central nuclei and the recovery of the cross-sectional area of muscle fibers. Therefore, our data systematically confirm the beneficial effect of hypoxia on the entire process of muscle regeneration caused by diabetes across molecular, cellular, and tissue levels. In diabetic skeletal muscle injury, hypobaric hypoxia produced concordant gains in vascular and myogenic indices, suggesting that coordinated modulation of oxygen partial pressure and barometric pressure can accelerate otherwise slow regeneration.

Hypobaric hypoxia reduces the atmospheric pressure and inspired oxygen concentration, inhibiting the degradation of HIF-1α by prolyl-hydroxylase. It also allows nuclear accumulation to drive transcription from hypoxia-response elements [24].

Skeletal muscle adapts to hypoxic conditions at biochemical and vascular levels. Consistent with prior studies, we observed increased HIF-1α protein after hypobaric hypoxia, supporting HIF-1α as the central transcriptional switch underlying its pro-regenerative effects [20,25].

At the early time point of day 7, hypobaric hypoxia enhanced and stabilized *HIF-1α* protein and upregulated VEGF and eNOS proteins. Transcripts of Kdr and Angpt2 were particularly responsive. Immunofluorescence revealed concurrent activation of Pax-7 and MyoD with high embryonic myosin heavy chain expression, both of which are indicators for nascent myotube formation. HIF-1α promotes VEGF release and VEGFR2 signaling in endothelial tip cells to enhance microvascular sprouting, and eNOS-derived nitric oxide improves microcirculatory flow and shear-dependent cues, increasing survival and directionality of early sprouts [26,27]. Because satellite cells reside in a relatively hypoxic niche and are responsive to HIF-1α during activation and differentiation, myogenic and vascular programs are initiated [28].

By day 14, hypobaric hypoxia increased Angpt1 transcripts and shifted the Angpt1-to-Angpt2 balance toward stabilization. Increases in Cdh5 and *Emcn* indicated recovery of endothelial adherens junctions and the glycocalyx, and CD31 architecture was clearer on histology. On the myogenic side, myogenin and mature myosin heavy chain rose as embryonic myosin heavy chain declined, which is consistent with the transition from early myotubes to mature fibers. Previous studies show that the Angpt1–Tie2 pathway maintains vascular quiescence and barrier integrity, stabilizes VE-cadherin junctions, reduces leakage, and improves perfusion [29]. Endomucin contributes to VEGFR2 endocytosis and signal regulation and is linked to cytoskeletal remodeling [30]. These changes create a low-inflammation, low-leak environment that supports fiber hypertrophy and matrix remodeling [31].

A strictly sequential model (vascular repair before myogenesis) does not explain the synchronous signals we observed: satellite cells secrete VEGF and receive angiocrine feedback from endothelium and immune cells, so vascular remodeling and myogenic differentiation proceed in parallel with stage-specific weighting, featuring early sprouting and microcirculatory gains, followed by junctional stabilization and fiber maturation [32,33]. Hypobaric hypoxia, as a systemic programmable hypoxia, stabilizes HIF-1α and activates the VEGF–eNOS cascade to initiate revascularization, then shifts toward barrier reconstruction centered on Angpt1, Angpt2, and Cdh5 [28,34,35,36,37]. On the myogenic axis, the trajectory advances from Pax-7- and MyoD-driven activation to myogenin and mature MyHC-driven maturation; in diabetes, baseline endothelial and myogenic defects heighten dependence on vascular stabilization and a low-inflammation milieu, so a parallel, phase-weighted framework best accounts for the multi-omic concordance on days 7 and 14 [1,38,39].

This study has limitations. First and foremost, while we demonstrate significant improvements in histological, molecular, and vascular markers of regeneration, direct functional outcomes—such as in vivo assessments of muscle contractile force, endurance, or locomotor recovery—were not measured. The analysis is limited to two time points and lacks early and long-term trajectories. Microvascular perfusion and barrier permeability were not directly quantified, and causal interrogation of HIF-1α, major receptors, *eNOS*, and angiopoietin–Tie2 signaling by genetic or pharmacologic means was not performed. In addition, future research will focus on the precise quantification of muscle fibers with central nuclei. This approach will provide a more reliable estimate of the number of new muscle fibers and facilitate a clearer correlation with molecular and functional indicators. Even so, protein, transcript, and histological evidence at both time points converge on parallel coupling with phase-specific emphasis, which is consistent with recent mechanistic and regenerative biology literature [1].

In summary, hypobaric hypoxia improved impaired regeneration after diabetic skeletal muscle injury. With no intervention, it conferred clear benefits for angiogenesis and myogenesis. These findings underscore the regenerative value of hypobaric hypoxia and provide useful insights for its application to the repair of diabetic skeletal muscle.

## 4. Materials and Methods

### 4.1. Animals

Male C57/BL6 mice (3 weeks old; Japan SLC, Shizuoka, Japan) were housed in cages with a 12 h light/dark cycle in a temperature-controlled setting (22 ± 2 °C). This work follows Kobe University Animal Experimentation Regulations and was approved by the Institutional Animal Care and Use Committee. All techniques followed the 1996 National Research Council-established NIH Guidelines for Laboratory Animal Care and USE.

A control group (NC = 5) was administered a normal-calorie diet, whereas a model group (DM = 35) received a high-fat diet (HFD-60, ORIENTAL YEAST, Osaka, Japan) for 12 weeks to induce type 2 diabetes. Mice were randomly assigned to these groups after one week of acclimatization. After 10 weeks on a high-fat diet, mice in the DM group were given streptozotocin at 50 mg/kg per day intraperitoneally for five days to induce DM and ablate pancreatic β-cells. Control animals received intraperitoneal injections of physiological saline.

Three groups of five DM mice were randomly assigned: uninjured, DM control group, muscle injury group (CTX), and muscle injury + hypobaric hypoxia group (H+CTX). The muscle injury model was established as previously described [40]. Cardiotoxin (10 μM Cytotoxin I, L8102 Latoxan, Portes-lès-Valence, France) was injected into the TA muscle to produce injury. To sedate mice, 4% isoflurane was inhaled, and hindlimb hair was removed. The skin was cleansed with 70% ethanol after hindlimb repair. Once the needle reached the intended injection site, the plunger was gently aspirated to ensure that no blood entered the syringe, thereby avoiding intravascular injection. Subsequently, CTX solution (50 μL) was injected into the TA muscle in an aseptic manner.

### 4.2. Glucose Tolerance Test

An IPGTT was performed seven days following the last STZ dose to confirm induction of diabetes (2.0 g/kg). The mice fasted for 16 h after changing bedding and were weighed the following day. Tail tips are clipped to sample blood for fasting glucose measurements (Glutest Neo, Sanwa Kagaku Kenkyuusyo, Nagoya, Japan). The number of mice determined the injection intervals, and intraperitoneal glucose was given to one animal. Blood glucose levels were tested 15 min following the first glucose dose administered to the mouse’s tail tip. Blood glucose was measured at 30, 60, and 120 min using the above method. To reduce increases in stress-induced blood glucose, mice were handled gently and quietly.

### 4.3. Hypobaric Hypoxia Chamber Treatment

Exposure to hypobaric hypoxia was performed using a hypobaric hypoxia chamber (O_2_ Room, Japan Press Bulk Industry, Shizuoka, Japan). Diabetic mice with muscle injury were placed in the chamber under conditions of 14.3% O_2_ at 0.7 atm for 8 h per day. The chamber was equipped with a computer-assisted system that precisely controlled both atmospheric pressure and oxygen concentration. The temperature was 22 °C, and the relative humidity was 45–55% throughout exposure.

After injury, the intervention group was immediately placed in the hypobaric hypoxia chamber, and tissue collection was performed on day 7 and day 14. Mice were slaughtered on days seven and fourteen. Following the inhalation of 4% isoflurane, compassionate euthanasia at 50 mg/kg sodium pentobarbital was injected intraperitoneally. Tibialis anterior (TA) muscles were dissected, weighed, and quickly frozen in dry ice/acetone. Materials were kept at −80 °C for future examinations.

### 4.4. Histological Analysis

Transverse tissue slices of the muscle mid-portion were cut into 12 μm thickness and placed on glass slides using a cryostat (CM-1510S, Leica Microsystems, Mannheim, Germany) at −25 °C.

To evaluate the myofiber cross-sectional area (CSA) and tissue remodeling, H&E staining was utilized. After 8 min in hematoxylin, sections were rinsed with tap water for 30 min, properly blotted, and counterstained with eosin for 1 min before rinsing for 30 min. After air-drying, slides were dehydrated in 70%, 80%, 90%, and 99% ethanol (1 min each), cleaned in xylene for 1 min, and covered with a neutral resin mounting solution. Bright-field photos were taken at a fixed magnification with similar exposure settings using a light microscope paired with a digital camera. Fibrosis was assessed using a modified Masson–Goldner trichrome staining kit (Merck, Tokyo, Japan) per the instructions. ImageJ (version 1.53a; National Institutes of Health, Bethesda, MD, USA) measured fibrosis (collagen-stained area %) and the myofiber cross-sectional area (CSA) in calibrated images, as previously described [41]. After 30 min in 4% paraformaldehyde, muscle slices were washed with 0.1 M PBS (pH 7.4) and blocked/permeabilized for 1 h in PBS with 3% bovine serum albumin and 1% Triton X-100. Sections were incubated overnight at 4 °C with primary antibodies used against Pax-7 (monoclonal, 1:200; sc-81648, Santa Cruz Biotechnology, Inc., Dallas, TX, USA), MyoD (polyclonal, 1:200; sc-760, Santa Cruz Biotechnology, Dallas, TX, USA) and CD31 (1:200 dilution, sc-376764; Santa Cruz, Biotechnology, Dallas, TX, USA). After washing, DyLight 549- or DyLight 488-conjugated secondary antibodies (1:1000; Jackson Immuno Research, West Grove, PA, USA) were applied for 1 h at room temperature in the dark, and nuclei were counterstained with DAPI (Invitrogen, Eugene, OR, USA). Images were acquired on a KEYENCE BZ-X800 microscopy system (KEYENCE, Osaka, Japan).

### 4.5. SDS–Polyacrylamide Gel Electrophoresis and Western Blot Analysis

Muscle tissues were homogenized using a lysis buffer (50 mM Tris-HCl, pH 8.0; 120 mM NaCl; 1% NP-40; 20 mM NaF; 1 mM EDTA; 1 mM EGTA; 15 mM Na-pyrophosphate; 30 mM β-glycerophosphate; 2 mM Na_2_VO_4_) and centrifuged at 1700× *g* for 10 min at 4 °C. To retain the values of the supernal protein, measurements were repeated. A Laemmli sample buffer was used with 50 mM Tris-HCl, pH 6.8, 2% SDS, 10% glycerol, 5% 2-mercaptoethanol, and 0.005% bromophenol blue, boiling with equal amounts of the supernatant at 80 °C for 10 min.

Using PVDF membranes, proteins (30 µg/lane) were analyzed after SDS-PAGE separation. A 1 h treatment with 5% skimmed milk in PBST reduced non-specific binding. The membranes, following blocking, were incubated with primary antibodies, including anti-VEGF (1:200; sc-7269, Santa Cruz Biochemistry, Dallas, TX, USA), MyoD (1:200; sc-760, Santa Cruz Biotechnology, Dallas, TX, USA), Pax-7 (1:200; sc-81648, Santa Cruz Biotechnology, Dallas, TX, USA), MyoG (1:300; sc-12732, Santa Cruz Biotechnology, Dallas, TX, USA), MyHC (1:500; 14-6503, eBioscience, San Diego, CA, USA), eMyHC (1:500; F1.652, Developmental Studies Hybridoma Bank, Iowa City, IA, USA), eNOS (1:1000; 6H2, #5880, Cell Signaling Technology, Danvers, MA, USA), HIF-1α(1:200; sc-10790, Santa Cruz Biotechnology, Dallas, TX, USA), and anti-GAPDH (1:1000; sc-32233, Santa Cruz Biochemistry, Dallas, TX, USA). Horseradish peroxidase-conjugated anti-rabbit or anti-mouse IgG (GE Healthcare, Waukesha, WI, USA) was used to detect bound primary antibodies. Chemiluminescent signals were subsequently generated with the Ez West Lumi reagent (ATTO, Tokyo, Japan) and captured using the LAS-1000 imaging platform (Fujifilm, Tokyo, Japan).

### 4.6. RNA Extraction and Quantitative RT–PCR Analysis

To collect spinal cord tissue samples, mice were euthanized 72 h post-injury. A 5 mm sample was promptly extracted, frozen in liquid nitrogen, and maintained at −80 °C until use. RNA was isolated from frozen spinal cord tissue using TRIzol™ Reagent (Invitrogen, Carlsbad, CA, USA) and homogenized using a Tissue Ruptor II (QIAGEN, Hilden, Germany) as per the manufacturer’s instructions. Total RNA was extracted using the TRIzol™ RNA isolation procedure (Invitrogen, Carlsbad, CA, USA) as per the manufacturer’s instructions. RNA concentration and purity were measured using a NanoDrop™ spectrophotometer(Thermo Fisher Scientific, Waltham, MA, USA). Only samples with an A260/A280 ratio between 1.8 and 2.1 were tested. A 20 µL reaction volume was used to synthesize first-strand cDNA from 500 ng of total RNA using the iScript™ cDNA Synthesis Kit (Bio-Rad, Hercules, CA, USA) as per the manufacturer’s instructions.

A qPCR was performed using the StepOne™ Real-Time PCR System (Applied Biosystems, Foster City, CA, USA), with the following cycle conditions: 3 min at 95 °C, 40 cycles of 10 s at 95 °C, and 30 s at 60 °C. In a 15 μL reaction, we added 8 μL cDNA, 1.5 μL 10× buffer, 0.3 μL 10 mM dNTPs, 1.5 μL 5 μM F+R primers, 3.58 μL nuclease-free water, 0.075 μL GoTaq^®^ DNA polymerase (Promega, Madison, WI, USA), and 0.045 μL 2× SYBR™ Green (Invitrogen, Carlsbad, CA, USA).

Normalization to the housekeeping gene *GAPDH* was performed to compute relative expression levels using the 2^−ΔΔCt^ technique. The primer sequences are presented as follows: Forward and reverse Emcn: AATACCAGGCATCGTGTCAGT and CTGATTCTCAGTCTTGTTCTGGG; Kdr: CAAACCTCAATGTGTCTCTTTGC (forward) and AGAGTAAAGCCTATCTCGCTGT (reverse); Cdh5: ATTGGATTTGGAACCAGATGC (forward) and CGCTTGACTTGATCTTGCC (reverse); Angpt1: GGGGGAGGTTGGACAGTAA (forward) and CATCAGCTCAATCCTCAGC (reverse); Angpt2: CCGCGGGCAAAATAAGTAGC (forward) and CACATGCGTCAAACCACCAG (reverse); GAPDH: CAGCAACTCCCACTCTTCCAC (forward) and TGGTCCAGGGTTTCTTACTC (reverse).

All responses were carried out in triplicate, with at least three biological duplicates examined for each group.

### 4.7. Statistical Analysis

Data are presented as the mean ± SEM. One-way ANOVA was performed to assess intergroup differences, followed by Tukey’s post hoc test for pairwise comparisons. Statistical significance was set at *p* < 0.05.

## Data Availability

The raw data supporting the conclusions of this article will be made available by the authors on request.

## Abbreviations

The following abbreviations are used in this manuscript:

ANOVA	Analysis of Variance
MyHC	Myosin Heavy Chain
eMyHC	Embryonic Myosin Heavy Chain
Pax-7	Paired Box Protein 7
Emcn	Endomucin
DAPI	4′,6-Diamidino-2-Phenylindole
Cdh5	Cadherin-5 (VE-cadherin)
VEGF	Vascular Endothelial Growth Factor
eNOS	Endothelial Nitric Oxide Synthase
Kdr	Kinase Insert Domain Receptor (VEGFR-2)
Angpt1	Angiopoietin-1
Angpt2	Angiopoietin-2
HE (H&E)	Hematoxylin and Eosin
HIF-1α	Hypoxia-Inducible Factor 1 Alpha
STZ	Streptozotocin
CTX	Cardiotoxin
DM	Diabetes Mellitus
MyoG	Myogenin
MyoD	Myogenic Differentiation Antigen
PBS	Phosphate-Buffered Saline
SDS-PAGE	Sodium Dodecyl Sulfate–Polyacrylamide Gel Electrophoresis
TA	Tibialis Anterior
IPGTT	Intraperitoneal Glucose Tolerance Test
CSA	Cross-Sectional Area
HFD	High-Fat Diet
PCR	Polymerase Chain Reaction
PFA	Paraformaldehyde
qRT-PCR	Quantitative Real-Time PCR
